# (*E*)-*N*′-(3,4-Dichloro­benzyl­idene)nicotino­hydrazide monohydrate

**DOI:** 10.1107/S1600536809034552

**Published:** 2009-09-05

**Authors:** Feng-Yu Bao, Ying-Xia Zhou, Hai-Yan Zhang, Su Hui

**Affiliations:** a Department of Applied Chemistry, College of Sciences, Henan Agricultural University, Zhengzhou 450002, People’s Republic of China

## Abstract

In the title compound, C_13_H_9_Cl_2_N_3_O·H_2_O, the 3,4-dichloro­benzene ring is nearly coplanar with the pyridine ring, making a dihedral angle of 4.78 (8)°. Inter­molecular O—H⋯O, O—H⋯N, N—H⋯O and weak C—H⋯O hydrogen bonding is present in the crystal structure.

## Related literature

For applications of Schiff base compounds, see: Kahwa *et al.* (1986 ▶); Santos *et al.* (2001 ▶).
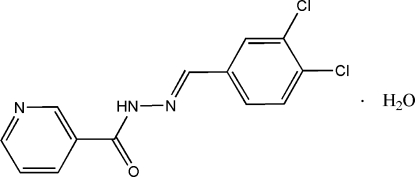


## Experimental

### 

#### Crystal data

C_13_H_9_Cl_2_N_3_O·H_2_O
*M* *_r_* = 312.15
Monoclinic, 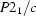

*a* = 8.2080 (3) Å
*b* = 12.3294 (4) Å
*c* = 13.7089 (4) Å
β = 91.522 (2)°
*V* = 1386.85 (8) Å^3^
*Z* = 4
Mo *K*α radiation
μ = 0.47 mm^−1^
*T* = 296 K
0.40 × 0.20 × 0.10 mm

#### Data collection

Bruker SMART CCD area-detector diffractometer
Absorption correction: multi-scan (*SADABS*; Bruker, 1998 ▶) *T*_min_ = 0.893, *T*_max_ = 0.954
20965 measured reflections
3032 independent reflections
2150 reflections with *I* > 2σ(*I*)
*R*_int_ = 0.042

#### Refinement

*R*[*F* ^2^ > 2σ(*F* ^2^)] = 0.036
*wR*(*F* ^2^) = 0.100
*S* = 1.02
3032 reflections
189 parameters
3 restraints
H atoms treated by a mixture of independent and constrained refinement
Δρ_max_ = 0.15 e Å^−3^
Δρ_min_ = −0.20 e Å^−3^

### 

Data collection: *SMART* (Bruker, 1998 ▶); cell refinement: *SAINT* (Bruker, 1998 ▶); data reduction: *SAINT*; program(s) used to solve structure: *SHELXTL* (Sheldrick, 2008 ▶); program(s) used to refine structure: *SHELXTL*; molecular graphics: *SHELXTL*; software used to prepare material for publication: *SHELXTL*.

## Supplementary Material

Crystal structure: contains datablocks global, I. DOI: 10.1107/S1600536809034552/xu2600sup1.cif

Structure factors: contains datablocks I. DOI: 10.1107/S1600536809034552/xu2600Isup2.hkl

Additional supplementary materials:  crystallographic information; 3D view; checkCIF report

## Figures and Tables

**Table 1:** Hydrogen-bond geometry (Å, °)

*D*—H⋯*A*	*D*—H	H⋯*A*	*D*⋯*A*	*D*—H⋯*A*
O1—H1*A*⋯O	0.85 (2)	1.995 (16)	2.8059 (19)	160 (2)
O1—H1*B*⋯N3^i^	0.85 (2)	2.079 (12)	2.909 (2)	166 (2)
N2—H2*A*⋯O1^ii^	0.86	2.00	2.842 (2)	165
C7—H7*A*⋯O1^ii^	0.93	2.55	3.314 (2)	140
C10—H10*A*⋯O1^ii^	0.93	2.39	3.304 (2)	167

Symmetry codes: (i) 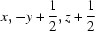; (ii) 
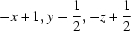
.

**Fig. 1.**
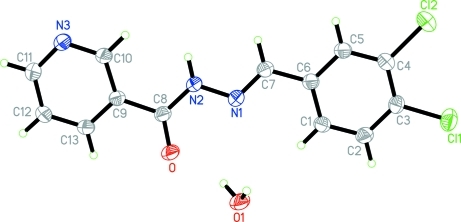
The molecular structure of the title compound. Displacement ellipsoids are drawn at the 30% probability level.

**Fig. 2.**
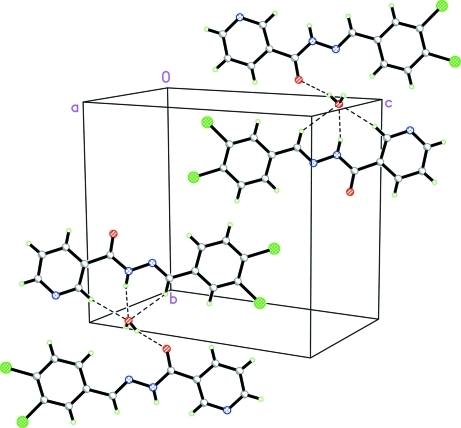
The unit cell packing diagram showing the intermolecular hydrogen bonding as dashed lines.

## supplementary crystallographic information

### Comment

The chemistry of Schiff bases has attracted a great deal of interest in recent years. These compounds play an important role in the development of various proteins and enzymes (Kahwa *et al.*, 1986; Santos *et al.*, 2001). As part of our interest in the coordination chemistry of Schiff bases, we have synthesized the title compound and report here its crystal structure.

The title molecule crystallizes in the E conformation (Fig. 1), with the N2—N1—C7—C6 torsion angle of 179.81 (15)°. The molecule structure is nearly planar, the dihedral angle between the 3,4-dichlorobenzene ring and the pyridine ring is 4.78 (8)°. The extensive intermolecular classic O—H···O, O—H···N, N—H···O and weak C—H···O hydrogen bonding is present in the crystal structure (Table 1 and Fig. 2).

### Experimental

Nicotinohydrazide (1 mmol, 0.137 g) was dissolved in ethanol (15 ml). The solution was stirred for several minitutes at 351 K, then the 3,4-dichlorobenzaldehyde (1 mmol, 0.175 g) in ethanol (8 ml) was added dropwise, and the mixture was stirred at refluxing temperature for 2 h. The solid product was isolated and recrystallized from methanol-water solution. Colourless single crystals were obtained after 3 d.

### Refinement

H atoms of water molecule are located in a difference Fourier map and refined isotropically, with O—H and H···H distances restrained to 0.85 (2) and 1.37 (2) Å. Other H atoms were positioned geometrically and refined as riding with C—H = 0.93 (aromatic) and N—H = 0.86 Å, *U*_iso_(H) = 1.2*U*_eq_(C,*N*).

### Crystal data

**Table:**

C_13_H_9_Cl_2_N_3_O·H_2_O	*F*(000) = 640
*M**_r_* = 312.15	*D*_x_ = 1.495 Mg m^−^^3^
Monoclinic, *P*2_1_/*c*	Mo *K*α radiation, λ = 0.71073 Å
Hall symbol: -P 2ybc	Cell parameters from 3887 reflections
*a* = 8.2080 (3) Å	θ = 2.5–27.0°
*b* = 12.3294 (4) Å	µ = 0.47 mm^−^^1^
*c* = 13.7089 (4) Å	*T* = 296 K
β = 91.522 (2)°	Block, colourless
*V* = 1386.85 (8) Å^3^	0.40 × 0.20 × 0.10 mm
*Z* = 4	

### Data collection

**Table:**

Bruker SMART CCD area-detector diffractometer	3032 independent reflections
Radiation source: fine-focus sealed tube	2150 reflections with *I* > 2σ(*I*)
graphite	*R*_int_ = 0.042
ω scans	θ_max_ = 27.0°, θ_min_ = 2.2°
Absorption correction: multi-scan (*SADABS*; Bruker, 1998)	*h* = −10→10
*T*_min_ = 0.893, *T*_max_ = 0.954	*k* = −15→15
20965 measured reflections	*l* = −17→17

### Refinement

**Table:**

Refinement on *F*^2^	Primary atom site location: structure-invariant direct methods
Least-squares matrix: full	Secondary atom site location: difference Fourier map
*R*[*F*^2^ > 2σ(*F*^2^)] = 0.036	Hydrogen site location: inferred from neighbouring sites
*wR*(*F*^2^) = 0.100	H atoms treated by a mixture of independent and constrained refinement
*S* = 1.02	*w* = 1/[σ^2^(*F*_o_^2^) + (0.0432*P*)^2^ + 0.3048*P*] where *P* = (*F*_o_^2^ + 2*F*_c_^2^)/3
3032 reflections	(Δ/σ)_max_ = 0.022
189 parameters	Δρ_max_ = 0.15 e Å^−^^3^
3 restraints	Δρ_min_ = −0.20 e Å^−^^3^

### Special details

**Table:**

Geometry. All e.s.d.'s (except the e.s.d. in the dihedral angle between two l.s. planes) are estimated using the full covariance matrix. The cell e.s.d.'s are taken into account individually in the estimation of e.s.d.'s in distances, angles and torsion angles; correlations between e.s.d.'s in cell parameters are only used when they are defined by crystal symmetry. An approximate (isotropic) treatment of cell e.s.d.'s is used for estimating e.s.d.'s involving l.s. planes.
Refinement. Refinement of *F*^2^ against ALL reflections. The weighted *R*-factor *wR* and goodness of fit *S* are based on *F*^2^, conventional *R*-factors *R* are based on *F*, with *F* set to zero for negative *F*^2^. The threshold expression of *F*^2^ > σ(*F*^2^) is used only for calculating *R*-factors(gt) *etc*. and is not relevant to the choice of reflections for refinement. *R*-factors based on *F*^2^ are statistically about twice as large as those based on *F*, and *R*- factors based on ALL data will be even larger.

### Fractional atomic coordinates and isotropic or equivalent isotropic displacement parameters (Å^2^)

**Table:**

	*x*	*y*	*z*	*U*_iso_*/*U*_eq_	
Cl1	−0.01836 (7)	0.35397 (5)	0.67808 (4)	0.07194 (19)	
Cl2	0.02126 (7)	0.10476 (5)	0.62576 (4)	0.0725 (2)	
N2	0.46330 (18)	0.25736 (12)	0.18259 (10)	0.0462 (4)	
H2A	0.4694	0.1892	0.1698	0.055*	
N1	0.38507 (18)	0.29373 (12)	0.26366 (10)	0.0471 (4)	
O	0.52773 (19)	0.42935 (10)	0.14108 (9)	0.0657 (4)	
C6	0.2369 (2)	0.25413 (14)	0.40566 (13)	0.0448 (4)	
C8	0.5303 (2)	0.33162 (14)	0.12366 (12)	0.0457 (4)	
C4	0.0960 (2)	0.20492 (15)	0.55082 (13)	0.0482 (4)	
C3	0.0803 (2)	0.31321 (16)	0.57470 (13)	0.0487 (4)	
C10	0.6343 (2)	0.18316 (14)	0.01178 (13)	0.0501 (4)	
H10A	0.5992	0.1314	0.0559	0.060*	
C2	0.1439 (2)	0.39176 (15)	0.51490 (14)	0.0527 (5)	
H2	0.1344	0.4646	0.5315	0.063*	
C9	0.6100 (2)	0.29128 (13)	0.03370 (12)	0.0420 (4)	
C13	0.6619 (2)	0.36685 (15)	−0.03277 (13)	0.0519 (5)	
H13A	0.6463	0.4404	−0.0214	0.062*	
C5	0.1740 (2)	0.17586 (15)	0.46627 (13)	0.0483 (4)	
H5A	0.1841	0.1029	0.4501	0.058*	
C7	0.3221 (2)	0.22160 (15)	0.31757 (13)	0.0480 (4)	
H7A	0.3301	0.1487	0.3011	0.058*	
C1	0.2212 (2)	0.36294 (14)	0.43093 (14)	0.0508 (5)	
H1	0.2632	0.4164	0.3909	0.061*	
O1	0.5421 (2)	0.52708 (11)	0.32595 (11)	0.0651 (4)	
N3	0.7054 (2)	0.14870 (12)	−0.06903 (11)	0.0564 (4)	
C11	0.7563 (2)	0.22393 (16)	−0.13096 (14)	0.0558 (5)	
H11A	0.8077	0.2014	−0.1871	0.067*	
C12	0.7366 (3)	0.33289 (16)	−0.11592 (14)	0.0575 (5)	
H12A	0.7731	0.3830	−0.1611	0.069*	
H1B	0.599 (3)	0.4849 (16)	0.3622 (14)	0.092 (9)*	
H1A	0.515 (3)	0.4919 (18)	0.2749 (11)	0.102 (10)*	

### Atomic displacement parameters (Å^2^)

**Table:**

	*U*^11^	*U*^22^	*U*^33^	*U*^12^	*U*^13^	*U*^23^
Cl1	0.0761 (4)	0.0845 (4)	0.0562 (3)	0.0074 (3)	0.0190 (3)	−0.0066 (3)
Cl2	0.0856 (4)	0.0646 (3)	0.0679 (3)	−0.0098 (3)	0.0148 (3)	0.0166 (3)
N2	0.0585 (9)	0.0371 (7)	0.0434 (8)	0.0010 (7)	0.0081 (7)	−0.0042 (6)
N1	0.0519 (9)	0.0439 (8)	0.0458 (8)	0.0025 (7)	0.0058 (6)	−0.0041 (7)
O	0.1088 (12)	0.0353 (7)	0.0541 (8)	0.0024 (7)	0.0196 (7)	−0.0034 (6)
C6	0.0423 (10)	0.0429 (9)	0.0492 (9)	−0.0002 (8)	0.0026 (7)	−0.0017 (8)
C8	0.0558 (11)	0.0386 (9)	0.0427 (9)	0.0027 (8)	0.0004 (8)	−0.0014 (7)
C4	0.0463 (11)	0.0494 (10)	0.0490 (10)	−0.0031 (8)	0.0013 (8)	0.0069 (8)
C3	0.0436 (10)	0.0562 (11)	0.0466 (10)	0.0029 (9)	0.0052 (8)	−0.0032 (8)
C10	0.0666 (12)	0.0379 (9)	0.0461 (10)	0.0021 (9)	0.0079 (8)	0.0030 (8)
C2	0.0546 (12)	0.0435 (10)	0.0603 (11)	0.0024 (9)	0.0084 (9)	−0.0050 (9)
C9	0.0466 (10)	0.0379 (9)	0.0414 (9)	−0.0006 (8)	−0.0016 (7)	−0.0014 (7)
C13	0.0654 (12)	0.0379 (9)	0.0527 (10)	−0.0046 (8)	0.0061 (9)	−0.0011 (8)
C5	0.0502 (11)	0.0411 (9)	0.0535 (10)	−0.0017 (8)	0.0018 (8)	−0.0016 (8)
C7	0.0508 (11)	0.0424 (10)	0.0509 (10)	−0.0001 (8)	0.0040 (8)	−0.0050 (8)
C1	0.0528 (11)	0.0413 (10)	0.0590 (11)	−0.0007 (8)	0.0119 (9)	0.0013 (8)
O1	0.1057 (13)	0.0364 (7)	0.0533 (8)	0.0038 (8)	0.0052 (8)	−0.0022 (7)
N3	0.0752 (11)	0.0435 (9)	0.0512 (9)	0.0039 (8)	0.0131 (8)	−0.0031 (7)
C11	0.0632 (13)	0.0558 (12)	0.0490 (10)	−0.0018 (10)	0.0123 (9)	−0.0050 (9)
C12	0.0703 (13)	0.0488 (11)	0.0541 (11)	−0.0098 (10)	0.0163 (10)	0.0021 (9)

### Geometric parameters (Å, °)

**Table:**

Cl1—C3	1.7255 (18)	C10—H10A	0.9300
Cl2—C4	1.7291 (18)	C2—C1	1.376 (2)
N2—C8	1.348 (2)	C2—H2	0.9300
N2—N1	1.3736 (19)	C9—C13	1.378 (2)
N2—H2A	0.8600	C13—C12	1.374 (3)
N1—C7	1.275 (2)	C13—H13A	0.9300
O—C8	1.229 (2)	C5—H5A	0.9300
C6—C5	1.383 (2)	C7—H7A	0.9300
C6—C1	1.392 (2)	C1—H1	0.9300
C6—C7	1.467 (2)	O1—H1B	0.85 (2)
C8—C9	1.497 (2)	O1—H1A	0.85 (2)
C4—C3	1.381 (3)	N3—C11	1.332 (2)
C4—C5	1.386 (2)	C11—C12	1.369 (3)
C3—C2	1.380 (3)	C11—H11A	0.9300
C10—N3	1.335 (2)	C12—H12A	0.9300
C10—C9	1.382 (2)		
			
C8—N2—N1	118.04 (14)	C13—C9—C8	118.00 (15)
C8—N2—H2A	121.0	C10—C9—C8	124.65 (15)
N1—N2—H2A	121.0	C12—C13—C9	119.66 (17)
C7—N1—N2	116.54 (15)	C12—C13—H13A	120.2
C5—C6—C1	118.97 (17)	C9—C13—H13A	120.2
C5—C6—C7	119.85 (16)	C6—C5—C4	120.70 (17)
C1—C6—C7	121.15 (16)	C6—C5—H5A	119.7
O—C8—N2	122.71 (16)	C4—C5—H5A	119.7
O—C8—C9	119.75 (16)	N1—C7—C6	119.73 (16)
N2—C8—C9	117.54 (15)	N1—C7—H7A	120.1
C3—C4—C5	119.74 (16)	C6—C7—H7A	120.1
C3—C4—Cl2	120.84 (14)	C2—C1—C6	120.31 (17)
C5—C4—Cl2	119.42 (14)	C2—C1—H1	119.8
C4—C3—C2	119.88 (16)	C6—C1—H1	119.8
C4—C3—Cl1	121.65 (14)	H1B—O1—H1A	107.2 (18)
C2—C3—Cl1	118.47 (15)	C11—N3—C10	117.30 (16)
N3—C10—C9	123.78 (17)	N3—C11—C12	123.14 (17)
N3—C10—H10A	118.1	N3—C11—H11A	118.4
C9—C10—H10A	118.1	C12—C11—H11A	118.4
C1—C2—C3	120.39 (17)	C11—C12—C13	118.75 (18)
C1—C2—H2	119.8	C11—C12—H12A	120.6
C3—C2—H2	119.8	C13—C12—H12A	120.6
C13—C9—C10	117.35 (16)		

### Hydrogen-bond geometry (Å, °)

**Table:**

*D*—H···*A*	*D*—H	H···*A*	*D*···*A*	*D*—H···*A*
O1—H1A···O	0.85 (2)	2.00 (2)	2.8059 (19)	160 (2)
O1—H1B···N3^i^	0.85 (2)	2.08 (1)	2.909 (2)	166 (2)
N2—H2A···O1^ii^	0.86	2.00	2.842 (2)	165
C7—H7A···O1^ii^	0.93	2.55	3.314 (2)	140
C10—H10A···O1^ii^	0.93	2.39	3.304 (2)	167

Symmetry codes: (i) *x*, −*y*+1/2, *z*+1/2; (ii) −*x*+1, *y*−1/2, −*z*+1/2.

## References

[bb1] Bruker (1998). *SMART*, *SAINT* and *SADABS* Bruker AXS Inc., Madison, Wisconsin, USA.

[bb2] Kahwa, I. A., Selbin, I., Hsieh, T. C. Y. & Laine, R. A. (1986). *Inorg. Chim. Acta*, **118**, 179–185.

[bb3] Santos, M. L. P., Bagatin, I. A., Pereira, E. M. & Ferreira, A. M. D. C. (2001). *J. Chem. Soc. Dalton Trans.* pp. 838–844.

[bb4] Sheldrick, G. M. (2008). *Acta Cryst.* A**64**, 112–122. 10.1107/S0108767307043930 18156677

